# Parental Communication, Engagement, and Support During the Adolescent Voluntary Medical Male Circumcision Experience

**DOI:** 10.1093/cid/cix970

**Published:** 2018-04-03

**Authors:** Kim H Dam, Michelle R Kaufman, Eshan U Patel, Lynn M Van Lith, Karin Hatzold, Arik V Marcell, Webster Mavhu, Catherine Kahabuka, Lusanda Mahlasela, Emmanuel Njeuhmeli, Kim Seifert Ahanda, Getrude Ncube, Gissenge Lija, Collen Bonnecwe, Aaron A R Tobian

**Affiliations:** 1 Johns Hopkins Center for Communication Programs, Baltimore, Maryland; 2 Johns Hopkins Bloomberg School of Public Health, Baltimore, Maryland; 3 Department of Pathology, Johns Hopkins University School of Medicine, Baltimore, Maryland; 4 Population Services International, Harare, Zimbabwe; 5 Department of Pediatrics, Johns Hopkins School of Medicine, Baltimore, Maryland; 6 Centre for Sexual Health and HIV/AIDS Research, Harare, Zimbabwe; 7 CSK Research Solutions, Dar es Salaam, Tanzania; 8 Centre for Communication Impact, Pretoria, South Africa; 9 Office of HIV/AIDS, Global Health Bureau, United States Agency for International Development, Washington, District of Columbia; 10 Ministry of Health and Child Care, Harare, Zimbabwe; 11 Ministry of Health, Community Development, Gender, Elderly and Children, Dar es Salaam, Tanzania; 12 National Department of Health, Pretoria, South Africa

**Keywords:** voluntary medical male circumcision, HIV, adolescents, parents, sub-Saharan Africa

## Abstract

**Background:**

Voluntary medical male circumcision (VMMC) is one of few opportunities in sub-Saharan Africa to engage male adolescents in the healthcare system. Limited data are available on the level of parental communication, engagement, and support adolescents receive during the VMMC experience.

**Methods:**

We conducted 24 focus group discussions with parents/guardians of adolescents (N = 192) who agreed to be circumcised or were recently circumcised in South Africa, Tanzania, and Zimbabwe. In addition, male adolescents (N = 1293) in South Africa (n = 299), Tanzania (n = 498), and Zimbabwe (n = 496) were interviewed about their VMMC experience within 7–10 days postprocedure. We estimated adjusted prevalence ratios (aPRs) using multivariable Poisson regression with generalized estimating equations and robust standard errors.

**Results:**

Parents/guardians noted challenges and gaps in communicating with their sons about VMMC, especially when they did not accompany them to the clinic. Adolescents aged 10–14 years were significantly more likely than 15- to 19-year-olds to report that their parent accompanied them to a preprocedure counseling session (56.5% vs 12.5%; *P* < .001). Among adolescents, younger age (aPR, 0.86; 95% confidence interval [CI], .76–.99) and rural setting (aPR, 0.34; 95% CI, .13–.89) were less likely to be associated with parental–adolescent communication barriers, while lower socioeconomic status (aPR, 1.37; 95% CI, 1.00–1.87), being agnostic (or of a nondominant religion; aPR, 2.87; 95% CI, 2.21–3.72), and living in South Africa (aPR, 2.63; 95% CI, 1.29–4.73) were associated with greater perceived barriers to parental–adolescent communication about VMMC. Parents/guardians found it more difficult to be involved in wound care for older adolescents than for adolescents <15 years of age.

**Conclusions:**

Parents play a vital role in the VMMC experience, especially for younger male adolescents. Strategies are needed to inform parents completely throughout the VMMC adolescent experience, whether or not they accompany their sons to clinics.

Adolescents aged 10–19 years comprise the majority of voluntary medical male circumcision (VMMC) clients in sub-Saharan Africa [1, 2]. VMMC is one of few opportunities in sub-Saharan Africa to engage male adolescents in the healthcare system [3]. Their parents/guardians, henceforth referred to as “parents,” play a key role in the adolescent VMMC experience, from the decision-making process to postprocedure wound care. Additionally, VMMC guidelines require written parental consent and consistently state that adolescents (<18 years of age) and their parents should receive comprehensive information regarding the benefits/risks of the VMMC procedure [4–7]. A better understanding of parents’ role in communication, engagement, and support as it pertains to VMMC is needed to respond effectively to the high demand for VMMC services among adolescents [8].

Studies in sub-Saharan Africa suggest that parent–adolescent communication regarding human immunodeficiency virus (HIV) and sexuality is more likely to occur if parents receive information to educate their child and perceive their child as ready to be exposed to such topics [9, 10]. Parental engagement in learning about VMMC and support during wound care has been shown to encourage uptake and improve wound care healing [11–14]. Linked to increasing preventive health behaviors among adolescents such as delayed sexual initiation and condom use, parent–adolescent communication has the potential to foster healthy adolescent development related to VMMC and beyond [15–22]. Limited data are available on the actual level of parental communication, engagement, and support that adolescents receive before, during, and after VMMC. This study uses mixed-methods approaches among parents and male adolescents aged 10–19 years to assess the extent to which parents engage in the adolescent VMMC experience in 3 sub-Saharan African countries.

## METHODS

### Study Setting and Design

Qualitative data were collected from parents and quantitative data were collected from male adolescents undergoing VMMC, either by surgery or elastic collar compression device (PrePex), in 14 clinic sites in South Africa, Tanzania, and Zimbabwe between June 2015 and September 2016. Discussions and interviews were conducted in Sesotho, isiZulu, or isiSwati (or in English if the participant preferred) in South Africa; KiSwahili in Tanzania; and Shona or Ndebele in Zimbabwe. Ethical approval was obtained from the Human Sciences Research Council in South Africa, Tanzania National Institute for Medical Research, Medical Research Council of Zimbabwe, and Johns Hopkins Bloomberg School of Public Health Institutional Review Board prior to data collection. All participants provided informed consent or assent (with parental consent).

### Qualitative Data Collection

Semistructured focus group discussions (FGDs) were conducted with parents recruited from communities surrounding clinic sites through VMMC mobilizers and/or trained research coordinators. Their adolescent sons had agreed to be circumcised or had been recently circumcised. Parent discussions explored attitudes regarding VMMC, expectations about the procedure and postprocedure concerns, and perceived self-efficacy in communicating and supporting their son. FGDs were audio recorded, transcribed, and translated into English for coding and analysis. The FGDs were conducted separately for mothers and fathers due to the sensitivity of discussion topics related to VMMC and adolescent sexuality.

### Quantitative Data Collection

Adolescents were recruited to participate on the day of their VMMC procedure through VMMC mobilizers and/or trained research coordinators. Structured interviews were conducted prior to preprocedure counseling, with a follow-up structured interview undertaken approximately within 7–10 days after the procedure. Adolescents who completed their follow-up interview are included in the analysis [23, 24].

#### Sociodemographics

Male adolescent age was assessed and coded as 10–14 vs 15–19 years [8]. Information on household-level assets and amenities generated a wealth index using principal components analysis, as previously described [23, 25], with scores categorized into tertiles to allow socioeconomic status comparisons within the sample. Other covariates assessed included setting (coded as urban, periurban, or rural); religion; and previous sexual experience (coded as no vs yes, inclusive of any mutual genital touching, oral, vaginal, or anal sex).

#### Perceived Parent Engagement and Adolescent Communication About Voluntary Medical Male Circumcision

Adolescents were asked at the follow-up interview whether their parent attended the preprocedure counseling session and how comfortable they had felt with their parent being present. Adolescents were also asked about their parents’ role in wound care. Potential reservations in communicating with one’s parents about VMMC were assessed at follow-up using 16 items adapted from the Parent-Adolescent Communication–Jaccard measure for the VMMC context [26]. Using a polychoric correlation matrix, principal components analysis and parallel analysis revealed that the 16-item parent–adolescent VMMC communication barriers scale was unidimensional. Factor loadings were estimated by a one-factor maximum likelihood model. Responses to each item were coded as 1 (“strongly disagree”) to 4 (“strongly agree”), and a mean 16-item composite score was calculated (Cronbach α = .92). Higher scores are reflective of greater reservation in communicating with one’s parent(s) about VMMC.

### Data Analysis

Qualitative data were analyzed by 2 coders using a 2-step process. All transcripts were first coded independently with predetermined areas of inquiry, and coders developed appropriate categories/subcategories as needed, followed by themes using coder-generated categories. All transcripts were double-coded, and any coding discrepancies were discussed between coders until a consensus was met. Qualitative data analyses were performed using ATLAS.ti, version 7 (Berlin, Germany).

We examined associations between male adolescents’ age group and all quantitative outcomes. Differences in the distribution of ordinal Likert responses by age group were assessed using the nonparametric Somers D test with Fisher’s Z-transformation and jackknife estimation of standard errors [27, 28]. Differences in binary response variables by age group were determined using modified Poisson regression with generalized estimating equations (GEE) and robust standard errors [29]. We further examined sociodemographic correlates of the parent–adolescent VMMC communication barriers score in the upper quintile (ie, the proportion of participants who had the highest reservations about communicating with their parents about VMMC). Adjusted prevalence ratios (aPRs) were estimated from a multivariable modified Poisson regression model with GEE and robust standard errors [29]. The final multivariable model included variables shown to be associated with the outcome after adjusting for age group and country (*P* < .15). Quantitative data analyses were performed using Stata SE software, version 14.2 (StataCorp, College Station, Texas).

## RESULTS

### Study Population

A total of 192 mothers (n = 97) and fathers (n = 95) participated in 24 FGDs in South Africa (n = 59), Tanzania (n = 60), and Zimbabwe (n = 73; 8 FGDs per country with 6–12 participants per FGD). A total of 1293 male adolescents in South Africa (n = 299), Tanzania (n = 498), and Zimbabwe (n = 496) were interviewed. Table 1 displays participant sociodemographic characteristics.

**Table 1.** Participant Characteristics by Country

Characteristic	All Countries	South Africa	Tanzania	Zimbabwe
**Qualitative FGDs (parents**)	**N = 192**	**n = 59**	**n = 60**	**n = 73**
Mean age, y (SD)	39.9 (11.5)	40.5 (10.1)	36.1 (10.1)	42.5 (12.8)
Gender				
Female	97 (50.5)	30 (50.8)	31 (51.7)	36 (49.3)
Male	95 (49.5)	29 (49.2)	29 (48.3)	37 (50.7)
Setting				
Urban	89 (46.4)	18 (30.5)	16 (26.7)	55 (75.3)
Periurban	43 (22.4)	13 (22.0)	30 (50.0)	0 (0.0)
Rural	60 (31.3)	28 (47.5)	14 (23.3)	18 (24.7)
Completed primary education				
No	31 (16.2)	18 (30.5)	5 (8.3)	8 (11.0)
Yes	161 (83.9)	41 (69.5)	55 (91.7)	65 (89.0)
**Quantitative survey (adolescents**)	**N = 1293**	**n = 299**	**n = 498**	**n = 496**
Mean age, y (SD)	13.5 (3.1)	13.8 (3.0)	12.0 (2.6)	14.7 (3.0)
Age group, y				
10–14	836 (64.7)	187 (62.5)	413 (82.9)	236 (47.6)
15–19	457 (35.3)	112 (37.5)	85 (17.1)	260 (52.4)
Setting				
Urban	696 (53.8)	107 (35.8)	233 (46.8)	356 (71.8)
Periurban	192 (14.9)	50 (16.7)	142 (28.5)	0 (0.0)
Rural	405 (31.3)	142 (47.5)	123 (24.7)	140 (28.2)
SES wealth score^a^				
Low	443 (34.3)	15 (5.0)	333 (66.9)	95 (19.2)
Moderate	419 (32.4)	94 (31.4)	151 (30.3)	174 (35.1)
High	424 (32.8)	183 (61.2)	14 (2.8)	227 (45.8)
Religion				
Christian	1231 (95.2)	269 (90.0)	480 (96.4)	482 (97.2)
Muslim	20 (1.6)	2 (0.7)	13 (2.6)	5 (1.0)
Traditional	13 (1.0)	0 (0.0)	0 (0.0)	13 (4.4)
Agnostic/other	25 (1.9)	4 (0.8)	9 (1.8)	12 (4.0)
Ever had sex				
No	1073 (83.0)	231 (77.3)	410 (82.3)	432 (87.1)
Yes	219 (16.9)	67 (22.4)	88 (17.7)	64 (12.9)
Most talked-to adult with personal matters^b^				
Mother	411 (31.9)	150 (50.8)	124 (25.0)	137 (27.6)
Father	301 (23.4)	42 (14.2)	113 (22.8)	146 (29.4)
Brother	199 (15.5)	37 (12.5)	71 (14.3)	91 (18.3)
Other family member^c^	198 (15.4)	48 (16.3)	56 (11.3)	94 (19.0)
Other adult^d^	30 (2.3)	11 (3.7)	8 (1.6)	11 (2.2)
Does not talk with adults about personal matters	148 (11.5)	7 (2.4)	124 (25.0)	17 (3.4)

Data are presented as No. (%) unless otherwise indicated.

Abbreviations: FGD, focus group discussion; SD, standard deviation; SES, socioeconomic status.

^a^SES was estimated based on household amenities and is only reflective of the distribution of wealth within the sample (tertiles).

^b^All countries, N = 1287; South Africa, n = 295; Tanzania, n = 496; Zimbabwe, n = 496.

^c^Other family members include uncles, aunts, grandmother, grandfather, nephews, cousins, sisters.

^d^Other adults include teachers, spiritual leaders, voluntary medical male circumcision community mobilizer.

### Parent–Adolescent Communication About Voluntary Medical Male Circumcision

FGDs conducted with parents revealed that beyond providing consent for VMMC, parents in all countries had discussed the benefits and/or risks with their son when choosing to undergo the procedure. In all countries, parents indicated that their son’s age determined whether they spoke to him about VMMC and what level of information they shared, especially with younger adolescents. Parents found that communicating with their son about VMMC was challenging, especially for those who did not accompany him to the clinic.

Coming alone [to the clinic]…[what they learn from VMMC becomes] their secret. They do not tell us that they were told that if they have sex with someone, they will be protected by 60%. If you asked them what they had been told, they would just laugh. You would try to ask him when he was alone at home … ‘So do they inject you first before they circumcise you? Were you awake or not? What did they use to cut?’ They don’t want to talk about it. They are shy. (female parent, FGD, Mt Darwin, Zimbabwe)

Parents who initially had limited VMMC knowledge noted that learning about the benefits of VMMC through their son, spouse, and/or reliable sources of VMMC information (such as religious or community leaders) convinced them VMMC was critical in protecting their son. However, parents in Zimbabwe cited fears in communicating about VMMC because of stigma related to HIV testing. In South Africa and Tanzania, most parents discussed being in support of HIV testing for their children.

I think one of the reasons we don’t encourage them [adolescent males] or talk to them about circumcision is because we fear that when they go to the clinic, they will be tested, and the result that comes out is reflective of ours. So it will make it difficult or undesirable for me to talk about it, because if the child gets tested and is found to be positive, he will come back to the parents looking for answers and blaming them for his condition. Since in most instances when the child is not circumcised, the peers will notice, and it will become the story of the town. They will say, ‘You see? His child was turned away.’ Because one of the reasons for being turned away is that the person could be HIV positive. (male parent, FGD, Bulawayo, Zimbabwe)

Despite challenges discussed by parents, a majority of adolescents reported a parent as the adult they talk to most about personal matters (mother [31.9%] or father [23.4%]; Table 1). The mean parent–adolescent VMMC communication barriers score (range, 1–4) for each item was <2.0 for each age group (Table 2). Among adolescents, younger age (aPR, 0.86; 95% confidence interval [CI], .76–.99) and rural setting (aPR, 0.34; 95% CI, .13–.89) were less likely to be associated with high perceived barriers to parental–adolescent communication about VMMC (Table 3). Lower socioeconomic status (aPR, 1.37; 95% CI, 1.00–1.87), being agnostic (or of a nondominant religion; aPR, 2.87; 95% CI, 2.21–3.72), and living in South Africa (aPR, 2.63; 95% CI, 1.29–4.73) were associated with greater perceived barriers to parental–adolescent communication about VMMC (Table 3). Overall, parent–adolescent communication regarding VMMC was found to be a dynamic multidirectional dialogue between parents and their sons, supplemented by information and support from other sources such as religious or community leaders.

**Table 2.** Perceived Barriers to Communication With Parent(s)/Guardian About Voluntary Medical Male Circumcision

Perceived Barriers to Parent–Adolescent Communication About VMMC	Factor Loading	Uniqueness	Overall (N = 1266)	10–14 y (n = 816)	15–19 y (n = 450)
I would be embarrassed talking to my parent(s)/guardian about VMMC.	0.5994	0.6407	1.76 (0.82)	1.75 (0.83)	1.82 (0.82)
My parent(s)/guardian would not want to answer my question about VMMC.	0.6996	0.5106	1.76 (0.71)	1.75 (0.74)	1.79 (0.63)
My parent(s)/guardian would only lecture me if I tried to talk to them about VMMC.	0.7061	0.5014	1.80 (0.76)	1.74 (0.72)	1.93 (0.80)
I don’t need to talk to my parent(s)/guardian about VMMC; I know what I need to know.	0.7406	0.4515	1.81 (0.75)	1.76 (0.74)	1.93 (0.77)
My parent(s)/guardian doesn’t know enough for me to want to talk with them about VMMC.	0.7532	0.4327	1.82 (0.73)	1.78 (0.73)	1.91 (0.73)
My parent(s)/guardian would not be honest with me if I talked with them about VMMC.	0.7671	0.4116	1.79 (0.74)	1.76 (0.75)	1.85 (0.72)
My parent(s)/guardian is too old to be able to relate to me about VMMC.	0.6884	0.5261	1.74 (0.69)	1.71 (0.71)	1.83 (0.64)
It would only make my parent(s)/guardian suspicious of me if I tried to talk to them about VMMC.	0.7949	0.3681	1.77 (0.73)	1.74 (0.75)	1.83 (0.70)
It would be difficult to find a convenient time and place to talk to my parent(s)/guardian about VMMC.	0.7597	0.4229	1.87 (0.79)	1.83 (0.78)	1.93 (0.80)
My parent(s)/guardian is just too busy to talk with me about VMMC.	0.7799	0.3917	1.79 (0.71)	1.76 (0.73)	1.85 (0.68)
My parent(s)/guardian would ask me too many personal questions if I tried to talk with them about VMMC.	0.6546	0.5715	1.90 (0.82)	1.88 (0.83)	1.97 (0.79)
My parent(s)/guardian doesn’t want to hear what I have to say when it comes to VMMC.	0.7878	0.3794	1.72 (0.65)	1.70 (0.67)	1.77 (0.62)
My parent(s)/guardian and I would only argue if we were to talk about VMMC.	0.7998	0.3603	1.66 (0.64)	1.64 (0.65)	1.72 (0.63)
My parent(s)/guardian would be embarrassed talking with me about VMMC.	0.7892	0.3772	1.71 (0.66)	1.69 (0.68)	1.77 (0.64)
I would have a difficult time being honest about my behavior with my parent(s)/guardian if we were to talk about VMMC.	0.7942	0.3692	1.75 (0.73)	1.70 (0.73)	1.86 (0.74)
My parent(s)/guardian would get angry if I tried to talk to them about VMMC.	0.7906	0.3749	1.62 (0.62)	1.60 (0.64)	1.66 (0.60)
Mean composite score (SD)			1.77 (0.49)	1.73 (0.50)	1.84 (0.47)
Median composite score (IQR)			1.88 (1.00–2.06)	1.81 (1.25–2.06)	2.00 (1.44–2.13)

Data are mean (SD) unless otherwise indicated. Responses to each question were coded as 1 (“strongly disagree”) to 4 (“strongly agree”). Factor loadings and unique variance estimates were calculated from a one-factor polychoric maximum likelihood model. A composite score was calculated by averaging all 16 items (score range: 1 [low] to 4 [high]); the scale had high internal consistency overall (α = .92) and within each country (α > .85). Higher scores are reflective of greater reservation in communicating with one’s parent(s)/guardian about VMMC.

Abbreviations: IQR, interquartile range; SD, standard deviation; VMMC, voluntary medical male circumcision.

**Table 3.** Sociodemographic Factors Associated With a High Adolescent–Parent Circumcision Communication Barriers Score^a^

Sociodemographic Characteristic	Proportion, % (n)	PR (95% CI)	*P* Value	aPR (95% CI)	*P* Value
Age group, y					
10–14	15.7 (128/816)	**0.84 (.74–.96**)	.010	**0.86 (.76–.99**)	.034
15–19	21.3 (96/450)	Ref		Ref	
Setting					
Urban	23.7 (163/687)	Ref		Ref	
Periurban	12.5 (24/192)	0.55 (.20–1.56)	.263	0.55 (.27–1.12)	.100
Rural	9.6 (37/387)	0.48 (.18–1.27)	.139	**0.34 (.13–.89**)	.028
SES wealth score					
Low	15.0 (65/433)	1.21 (.87–1.71)	.261	**1.37 (1.00–1.87**)	.047
Moderate	17.0 (70/413)	0.94 (.75–1.17)	.575	0.95 (.81–1.11)	.529
High	21.0 (87/414)	Ref		Ref	
Religion					
Christian	17.0 (205/1206)	Ref		Ref	
Muslim	15.0 (3/20)	1.24 (.60–2.55)	.561	1.13 (.41–3.18)	.805
Traditional	23.1 (3/13)	1.00 (.21–4.71)	.999	1.14 (.26–4.99)	.863
Agnostic/other	56.5 (13/23)	**3.02 (2.07–4.39**)	<.001	**2.87 (2.21–3.72**)	<.001
Ever had sex					
No	16.4 (173/1052)	Ref		…	
Yes	23.8 (51/214)	1.28 (.97–1.69)	.086	…	
Country					
Tanzania	12.2 (60/491)	Ref		Ref	
Zimbabwe	19.2 (95/496)	2.37 (.89–6.31)	.084	1.80 (.93–3.46)	.090
South Africa	24.7 (69/279)	**2.63 (1.13–6.11**)	.025	**2.63 (1.29–4.73**)	.008

Prevalence ratios and 95% CIs were estimated from modified Poisson regression models with generalized estimating equations and robust variance estimation to account for clustering at the facility level. The final multivariable model included variables shown to be associated with the outcome after adjusting for age group and country (*P* < .15). Predictors shown to be statistically significant in univariable and multivariable analysis are shown in bold (*P* < .05).

Abbreviations: aPR, adjusted prevalence ratio; CI, confidence interval; PR, prevalence ratio; SES, socioeconomic status.

^a^Greater than upper quintile.

### Parental Engagement in Voluntary Medical Male Circumcision Uptake

While a few parents in all countries felt the decision to undergo VMMC was their son’s choice, most parents indicated it was either a decision they made together with their son (and sometimes with their spouse, especially for mothers) or a decision they made for their son. In some instances, particularly in Zimbabwe and South Africa, both mothers and fathers emphasized that VMMC was a matter for fathers or male heads of households to address. One mother in South Africa expressed fear of being blamed if her son was injured as a result of undergoing VMMC against her spouse’s wishes.

Parents in all countries expressed that it was important to accompany younger male adolescents to the clinic to provide emotional and wound care support. Some parents experienced challenges in being able to attend, such as resistance from older adolescents. Parents cited wanting to ease their son’s fears, help him understand the benefits/risks of VMMC and wound care, and make sure he followed through with the procedure, particularly with younger adolescents.

I would like to be there during the counseling as the parent of a child who’s 10–12 years old. I would like to be involved because the child is still young, and he forgets easily. I will help to recall some of the information. (male parent, FGD, Ermelo, South Africa)

**Figure 1.**
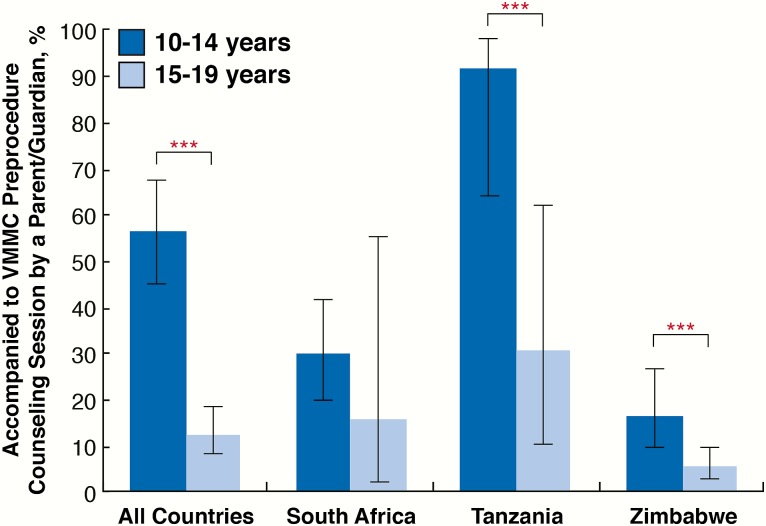
Parental attendance at voluntary medical male circumcision (VMMC) counseling services. Differences in responses by age group were determined by modified Poisson regression models with generalized estimating equations (GEE) and robust variance estimation. Error bars are 95% confidence intervals estimated by Taylor series linearization. ***P < .001.

Compared to 15- to 19-year-olds, 10- to 14-year-old males were significantly more likely to report that their parent accompanied them to a preprocedure counseling session on the day of the procedure (56.5% vs 12.5%; *P* < .001; Figure 1). Resistance from older adolescents was evident; a greater portion of 15- to 19-year-olds reported being a little or very uncomfortable with their parent attending their preprocedure counseling session, compared with 10- to 14-year-olds (16.3% vs 5.7%; *P* = .005). In all countries, direct parent engagement in VMMC uptake was mostly limited to discussing benefits/risks prior to the procedure with their sons and providing consent.

### Perceived Parental Support After Voluntary Medical Male Circumcision Procedures

Compared to the wound care support provided to adolescents <15 years of age, parents found it more difficult to be involved in wound care for older adolescents. Instead, they provided verbal support in the form of daily hygiene reminders and reduction of chores. For parents who were unable to accompany their son to the clinic, some expressed the need for information given by phone or through materials on wound care for their son to take home. Overall, parents encountered resistance in providing support (eg, bandage changing, cleaning the wound with salt solution) to sons across all age groups, citing sons’ embarrassment as one of the major reasons.

I tell [my grandson], ‘I would like to bathe you’; he refuses to remove his underpants. So when I bathe him, I scrub the rest of the body but he will be wearing his underpants. So since he got circumcised, he no longer wants me to touch or wash his privates. He actually says, ‘Granny, I don’t want you to see my privates. Scrub the other parts but I will finish off the remaining sections on my own.’ (female parent, FGD, Mt Darwin, Zimbabwe)

In all countries, parents discussed being divided on whether to talk with their son about sex following VMMC. Many felt it was important to talk about refraining from sex after the procedure and felt confident communicating that message to ensure their son would heal properly. However, when prompted, only a few parents had begun discussing the topic of sexuality with their sons, while others were waiting until after the VMMC procedure or until their son reached a certain age. Across all 3 countries, parents rarely discussed sexual health messages related to condom use, safer sex practices, and HIV (which are part of the VMMC minimum package of services [30]) with older adolescents and not at all with adolescents 10–12 years old. They felt ashamed, embarrassed, or ill-equipped to discuss sexuality and feared that talking about sex would encourage their sons to engage in it. In all sites, most parents felt that providers could discuss sex as related to VMMC with sexually active male adolescents and often assumed younger adolescents (<15 years of age) were not sexually active.

I don’t see a necessity in talking about not having sex because he doesn’t have it at all. I’ll just tell him that you are going to circumcise now, and you are going to stay for 6 weeks so that you can heal. That is what is important to him. Other things that you will tell him, it won’t help him now. I will tell him when he is ready. (female parent, FGD, Ermelo, South Africa)

**Figure 2.**
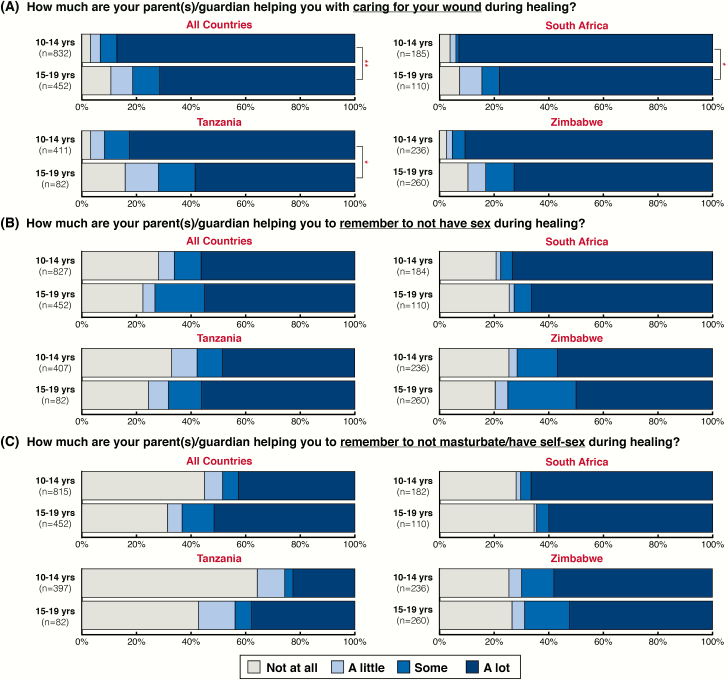
Perceived parental support during the postprocedure wound healing period.

Similarly, male adolescents across all countries perceived their parents as having supported them in caring for their wound (mean, 3.6 [standard deviation {SD}, 0.8]; n = 1284). Adolescents 10–14 years old were significantly more likely than 15- to 19-year-olds to perceive this support (mean, 3.8 [SD, 0.7] vs 3.4 [SD, 1.0]; *P* < .01; Figure 2A). Although male adolescents generally noted having support from parents regarding reminders to abstain from sex (mean, 3.0 [SD, 1.3]; n = 1279) and masturbation/self-sex (mean, 2.6 [SD, 1.4]; n = 1267) postprocedure, no associated age difference was seen when accounting for the clustered study design (Figure 2B and 2C). The distribution of scores for male adolescents’ perceived support from their parents appeared lower regarding matters of sex/masturbation than wound care (Figure 2).

Differences in the distribution of responses of each item (range, 1–4) were determined by nonparametric Somers D tests corrected for clustering at the facility level. *P < .05; **P < .01.

## DISCUSSION

Findings from both the parent and male adolescent perspectives demonstrate the vital role that parents play in helping sons understand the benefits/risks of VMMC, engaging in VMMC uptake, and ensuring proper healing postprocedure. However, the level of parental support appears to be age-dependent, with parents of younger male adolescents more involved. Male adolescents who were older and those from lower socioeconomic backgrounds reported greater perceived barriers in communicating with their parents about VMMC. Discussions with parents indicate major challenges and gaps in communication with their sons during all stages of the adolescent VMMC experience.

Parents in the study did not support discussions of HIV risk and sexual health information during the counseling session, particularly for those they perceived as not sexually active, such as younger adolescents. Parents feared that early exposure to information about sexual health would promote early sexual debut or risky sexual behaviors, despite evidence to the contrary [31]. A study of parent–child communication in Tanzania found parents to be constrained by cultural and gender norms and their lack of appropriate knowledge regarding HIV, modern contraception, and condoms [32, 33]. Of notable concern is the finding that parents’ fears related to discussing HIV and undergoing HIV testing (due to stigma related to testing HIV positive) may be a barrier to VMMC uptake by male adolescents. As described elsewhere [34], parents need assistance, particularly with respect to their younger sons, with talking about or supporting others talking with their sons about topics beyond abstinence that are pertinent to promoting HIV testing and prevention behaviors. Reducing parents’ fears related to HIV testing may also have implications for reaching vulnerable male adolescents, such as those living with HIV, for VMMC uptake and, subsequently, HIV testing and services [35].

Study findings indicate that although parents are engaged in helping their sons make decisions about VMMC, they are not always present for the procedure to receive detailed benefit/risk information and specific instructions about wound care. These findings are consistent with other studies showing inconsistent parental engagement for VMMC across high-burden focus countries. For instance, in Tanzania, where VMMC guidelines recommend involving parents during postprocedure wound care [1, 6], parents are included in group education and even individual counseling for young adolescents. In Zimbabwe, community mobilizers obtain written consent from parents not able to accompany their children to VMMC sites [1]. Strategies are needed to properly disseminate complete information to parents throughout the VMMC adolescent experience, irrespective of whether or not they accompany their sons to clinics. This might include holding separate information sessions for parents and developing tool kits and printed materials to improve parents’ understanding of VMMC during all stages of the VMMC experience, especially on postprocedure wound care, which is critical to the prevention of adverse events [14].

This study has some limitations. The cross-sectional design precludes drawing causal conclusions. Selection bias may be present, as this study was limited to adolescents who completed both a pre- and postprocedure interview. In addition, parents of adolescents who are going to or have undergone VMMC were selected based on convenience sampling and were not necessarily parents of interviewed male adolescents. Similar findings across countries, however, support the generalizability of these results.

Aside from VMMC, few opportunities exist for male adolescents in sub-Saharan Africa to formally connect with the healthcare system. Addressing these gaps in communication is critical, as VMMC is not only an opportunity to provide adolescent males with the care they need but, if well organized, can provide parents with the tools to help adolescents address their immediate needs related to VMMC and other health issues.

## Notes

***Acknowledgments.*** We are grateful to the adolescent participants in this study for sharing their perspectives and experiences. The authors also thank the VMMC facility managers, community mobilizers, and providers for their support, and acknowledge the contributions of the Technical Advisory Group for the adolescent VMMC assessment and their guidance throughout the study. We are privileged to work with this group of dedicated professionals from the US President’s Emergency Plan for AIDS Relief (PEPFAR), Centers for Disease Control and Prevention, US Department of Defense, World Health Organization, United Nations Children’s Fund, and the Bill & Melinda Gates Foundation. Thanks also go to the Human Sciences Research Council in South Africa, CSK Research Solutions in Tanzania, PSI/Zimbabwe, and the Centre for Sexual Health & HIV/AIDS Research in Zimbabwe for their assistance with data collection. The authors also appreciate the assistance of Meaghen Murphy with copy editing and Maria Elena Figueroa with project startup.

***Financial support.*** This work was supported by USAID with PEPFAR funding (cooperative agreement number AID-OAA-A-12-00058) to the Johns Hopkins Center for Communication Programs, and co-funded by UK Department of International Development through the Integrated Support Program in Zimbabwe.

***Supplement sponsorship*** This article appears as part of the supplement “Adolescent Voluntary Medical Male Circumcision: Vital Intervention Yet Improvements Needed,” sponsored by Johns Hopkins University.

***Potential conflicts of interest.*** All authors: No reported conflicts of interest. All authors have submitted the ICMJE Form for Disclosure of Potential Conflicts of Interest. Conflicts that the editors consider relevant to the content of the manuscript have been disclosed.
